# Long-term effects of anti-*N*-methyl-d-aspartate receptor encephalitis on quality of life

**DOI:** 10.3389/fneur.2023.1170961

**Published:** 2023-05-18

**Authors:** Satoshi Hirose, Makoto Hara, Yuki Yokota, Hideto Nakajima

**Affiliations:** Division of Neurology, Department of Medicine, Nihon University School of Medicine, Tokyo, Japan

**Keywords:** anti-N-menthyl-D-aspartate receptor encephalitis, long-term outcome, patient-oriented outcome, quality of life, sequelae

## Abstract

**Background:**

Patients with anti-*N*-methyl-d-aspartate receptor encephalitis (NMDARE) usually achieve neurologically favorable outcomes in the post-acute-phase. Even when motor function recovers, many patients experience numerous non-motor sequelae and cannot resume their pre-NMDARE lives even years later. Additionally, the needs of patients with NMDARE may impose a severe caregiver burden. Unfortunately, few studies have comprehensively examined patients recovering from NMDARE. We investigated the long-term effects of NMDARE on patients’ quality of life (QOL).

**Methods:**

Data collected via structured self-reported questionnaires included clinical features, long-term outcomes, and QOL. These questionnaires were administered to adult members of the Japanese Anti-NMDARE Patients’ Association. We used the NeuroQOL battery to assess QOL in physical, mental, and social domains. Raw NeuroQOL scores were converted to T-scores for comparison with controls.

**Results:**

Twenty-two patients completed the questionnaire. The median interval between disease onset and questionnaire response was 78  months. Forty-six percent of patients reported persistent sequelae, with only 73% able to resume prior work/school activities. Although patients’ Global QOL was similar to controls, patients with NMDARE had significantly worse social QOL. Patients with worse social QOL had more frequent sequelae than those with better social QOL. Furthermore, patients with persistent sequelae had significantly worse Global QOL than those without sequelae and controls.

**Conclusion:**

Patients with NMDARE had worse social QOL than controls. Given the adverse effects of disease sequelae on QOL, treatment strategies that minimize sequelae during the acute-phase may improve patients’ QOL, even years post-disease onset.

## 1. Introduction

Anti-*N*-methyl-d-aspartate receptor encephalitis (NMDARE) is the most common type of autoimmune encephalitis (1, 2). Acutely, NMDARE causes severe and multiple neurologic symptoms (1, 2). Most patients with NMDARE generally respond well to immunotherapy and achieve neurologically favorable outcomes (3). However, in contrast to the substantial motor recovery demonstrated by these patients, adverse sequelae—including cognitive (4), sleep (5), and psychosocial disorders (6, 7)—often persist many years post-disease onset (8). In addition, pediatric patients with NMDARE often demonstrate fatigue, potentially worsening quality of life (QOL) (9). NMDARE sequelae may impose a severe caregiver burden (10) and prolong the time needed to return to baseline work or school pursuits (6, 8), even years post-disease onset. Based on these facts, there is an urgent and unmet need for comprehensive outcome measures encompassing more than motor function, including cognition, psychosocial function, activities of daily living, autonomy, and QOL (11, 12).

The NeuroQOL is a clinically relevant and psychometrically robust QOL assessment tool for adults and children with neurologic disorders (13, 14). In addition, the NeuroQOL comprehensively captures physical, mental, and social health experiences to assist researchers studying neurologic disorders. We examined long-term outcomes in patients with NMDARE and assessed the long-term effects on QOL using the NeuroQOL battery.

## 2. Materials and methods

### 2.1. Protocol approval and patient consent

Participants were adults with NMDARE who belonged to the Japanese Anti-NMDARE Patients’ Association (Supplementary Methods) in July 2022. Written informed consent for study participation was obtained from the patients or their family members. The study was approved by the Nihon University School of Medicine’s ethics committee.

### 2.2. Questionnaires

The study questionnaire consisted of two parts. Part 1 assessed demographics, clinical features, and long-term outcomes; part 2 assessed QOL. Part 1 collected data on (1) age, (2) sex, (3) onset date, (4) presence of associated tumors, (5) neurological status and outcomes, as determined by the modified Rankin Scale (mRS) score (15), (6) intensive care unit (ICU) stays, (7) the need for mechanical ventilation, (8) presence of neuropsychiatric sequelae or personality changes, (9) return to previous work or school, (10) presence of any disability or handicap affecting daily life at home, and (11) presence of clinical relapses. Detailed questionnaire information is included in the Supplementary Methods.

Part 2 evaluated QOL using the NeuroQOL assessment for adults, a clinically relevant and psychometrically robust QOL assessment tool that comprehensively captures patients’ physical, mental, and social experiences (13, 14). The NeuroQOL includes 12 domain “short forms” consisting of (1) upper extremity function (v1.0), (2) lower extremity function (v1.0), (3) fatigue (v1.0), (4) sleep disturbance (v1.0), (5) depression (v1.0), (6) anxiety (v1.0), (7) stigma (v1.0), (8) positive affect and well-being (v1.0), (9) emotional and behavioral dyscontrol (v1.0), (10) cognitive function (v2.0), (11) satisfaction with social roles and activities (v1.1), and (12) ability to participate in social roles and activities (v1.0).

### 2.3. Inclusion criteria

Participants aged ≥ 18 years with an interval of at least 24 months between disease onset and the questionnaire response were included in the study.

### 2.4. Analysis of QOL

Raw NeuroQOL scores for each of the 12 domains were converted to standardized T-scores using Scoring Manual Version 3.0, available at: https://www.healthmeasures.net/images/neuro_qol/User_and_scoring_guides/Neuro-QOL_Scoring_Manual_26April2021_FINAL.pdf. T-score transformation allowed us to compare domain-specific QOL between patients and controls (13). T-scores of the reference controls averaged 50; therefore, we could determine if patients had significantly worse QOL than controls by comparing the patients’ T-scores to 50 (16).

To assess patients’ overall QOL, we merged T-scores from 12 domains into a single, patient-specific “Global QOL” score, as detailed in Supplementary Methods. Briefly, the 12 NeuroQOL domains were classified into “positive categories,” where higher T-scores indicate better QOL, or “negative categories,” where higher T-scores indicate worse QOL (13). Next, we transformed negative category T-scores into “inverted T-scores” so that higher scores indicated better QOL. Then, we averaged the positive category and inverted negative category T-scores across various physical, mental, and social domains (Supplementary Figure 1). Finally, we defined each patient’s “Global QOL” as the average of physical, mental, and social domain scores (Supplementary Figure 1).

### 2.5. Statistics

Statistically significant differences in NeuroQOL T-scores between patients and controls were detected using the Wilcoxon signed-rank test. Significant differences in clinical features and long-term outcomes between patients with better and worse QOL were tested using Fisher’s exact test for categorical data and the Mann–Whitney *U*-test for numerical data. The Mann–Whitney *U*-test determined between-group differences in NeuroQOL T-scores in patients with and without sequelae. *P*-values < 0.05 were considered statistically significant.

## 3. Results

### 3.1. Demographics

**Figure 1:**
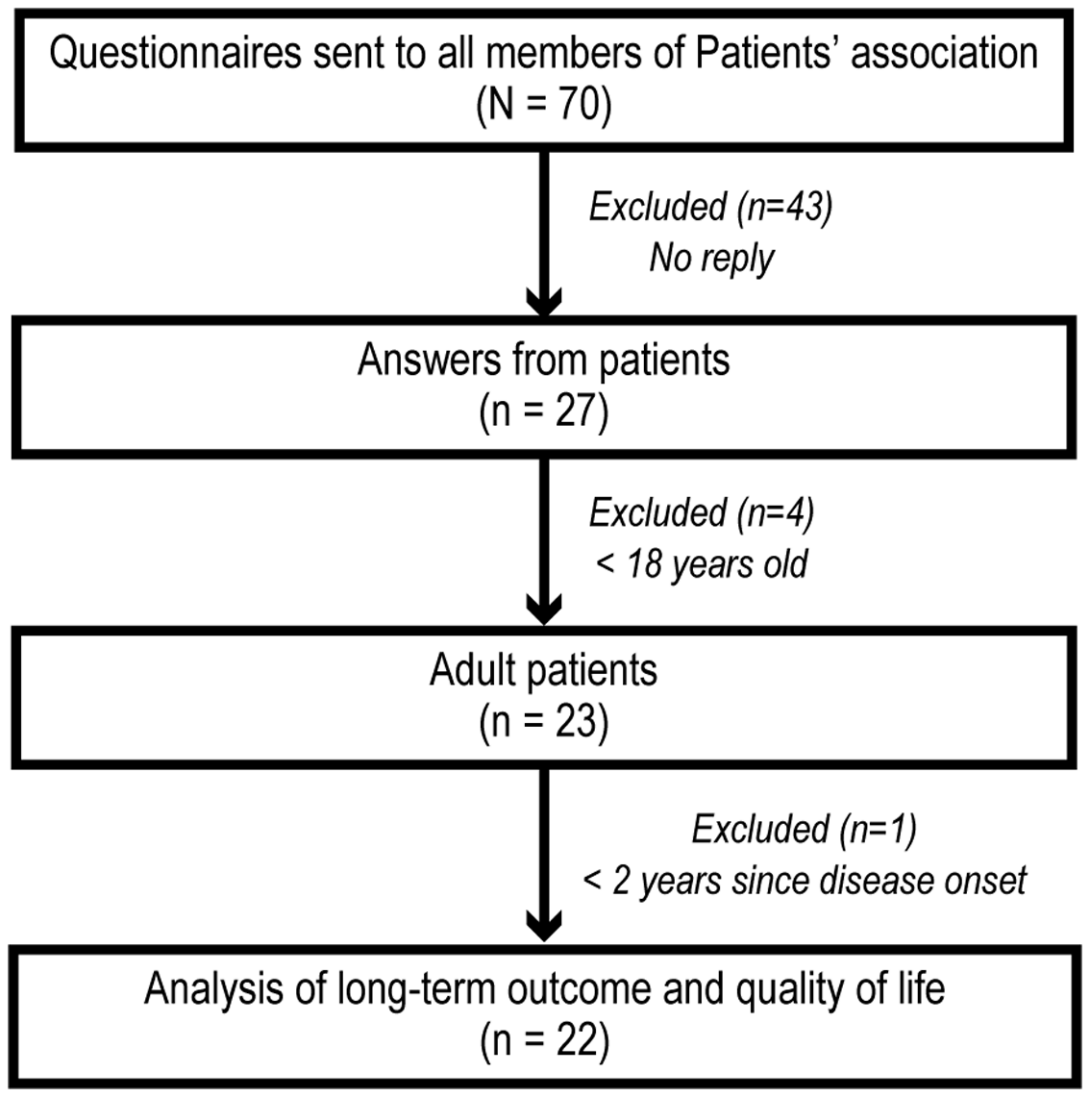
Patient selection and analysis flowchart. Questionnaires were sent to all 70 Japanese Anti-NMDARE Patients’ Association members. Answers were obtained from 27 patients. Four patients were excluded because they were  <18  years old. Another patient was excluded because the interval between disease onset and questionnaire response was <2  years. We analyzed the long-term outcomes and QOL scores for the remaining 22 patients.

We sent questionnaires to all 70 members of the Anti-NMDARE Patient Association in July 2022; 27 patients returned their questionnaires (response rate 39%; Figure 1). Four patients were excluded because they were younger than 18 years, and another patient was excluded because the interval between disease onset and questionnaire completion was less than 2 years. We analyzed the remaining 22 patients’ long-term outcomes and QOL (Figure 1). All were previously diagnosed with NMDARE by cell-based assays using NR1/NR2–transfected HEK293 cells. Demographics data are included in Supplementary Table 1. The median age at answer date was 28 (range, 19–57) years and most (*n* = 19; 86%) were female. The median interval between onset and answer dates was 77.5 (range, 26–162) months.

### 3.2. Clinical features and long-term outcomes

The 22 patients’ clinical features and long-term outcomes are included in Supplementary Table 1. Ten patients (46%) had associated tumors, all female. The median mRS score at the worst status was 5 (range, 2–5) but eventually improved to 0 (0–5). Of the 20 patients who responded to the question regarding ICU stay, 14 (70%) reported being admitted to ICUs. Eleven (50%) patients required mechanical ventilation. Persistent sequelae were reported by 10 (46%) patients and included memory disorders (36%), psychotic symptoms (9%), movement disorders (e.g., mild dystonia; 9%), motor paralysis (including sequelae derived from critical illness neuropathy; 9%), urination disorders (9%), speech disorders (5%), and taste disturbances or *dysgeusia* (5%). No patients reported seizures or olfactory impairment. Nine (41%) patients felt their personalities changed over the course of their illnesses. Sixteen (73%) patients returned to prior work/school activities, and 17 (77%) eventually functioned independently at home. Five (23%) patients showed clinical relapses; once in four patients, and three times in another patient.

### 3.3. Comparison of QOL between patients and controls

We obtained NeuroQOL battery responses across 12 domains from all 22 patients. Raw domain scores were converted to standardized T-scores as previously described (13). Patients had significantly worse QOL than controls for satisfaction with social roles and activities (*p* = 0.042) and ability to participate in social roles and activities (*p* = 0.028; Table 1). The other 10 domain T-scores were not significantly worse than controls. T-scores in all 12 domains are included in Supplementary Table 2. We observed no significant differences for Global QOL between patients and controls (Table 1).

**Table 1:** Neuro-QOL T-scores of NMDARE patients compared with controls.

Neuro-QOL domain	Median (range)	*p*-value^†^ (comparison with controls)
Global QOL	52.3 (24.6–62.2)	0.615
Satisfaction with social roles and activities	47.9 (32.6–60.5)	0.042^*^
Ability to participate in social roles and activities	45.8 (24.1–60.2)	0.028^*^

NMDARE, anti-N-methyl-d-aspartate receptor encephalitis.

† Statistically significant differences in NeuroQOL T-scores between patients and controls were detected using the Wilcoxon signed-rank test, ^*^p < 0.05.

### 3.4. Analyses of the association between QOL and sequelae

Based on the results that patients had significantly worse QOL in the two social domains (namely, satisfaction with social roles and activities, and ability to participate in social roles and activities), we compared patients with better and worse social domain QOL scores. We divided patients into two groups according to the control group’s average T-score (i.e., 50). Eight patients demonstrated better (i.e., ≥50) social domain QOL scores than controls; 14 had worse (i.e., <50). As shown in Table 2, there were significantly more patients with persistent sequelae among patients with worse compared to better social domain QOL scores (71% vs. 0%, *p* = 0.002). Among the various sequelae, memory disorders were most frequently reported (36%). Significantly more patients in the worse QOL group demonstrated impaired memory than the better QOL group (57% vs. 0%, *p* = 0.018). No significant between-group differences were found for any other factor (Table 2).

**Table 2:** Comparison of clinical features and long-term outcomes between patients with better/worse QOL in social domains.

	Better QOL (*n* = 8)	Worse QOL (*n* = 14)	*p*-value^†^ (comparison)
Age, median (range)	26.5 (20–37)	31.5 (19–57)	0.330
Female (%)	87.5	85.7	1.000
Duration since onset, months, median (range)	67 (26–153)	90 (28–162)	0.714
Tumor (%)	37.5	50.0	0.675
Stay in ICU^#^ (%)	42.9	84.6	0.122
Use of ventilator (%)	25.0	64.3	0.183
Favorable mRS (≤2) at present (%)	100.0	71.4	0.254
Return to previous work/school life (%)	100.0	57.1	0.051
Self-reliance at home life (%)	100.0	64.3	0.115
Sequelae (%)	0	71.4	0.002*
Personality change (%)	25.0	50.0	0.380
Relapse (%)	25.0	21.4	1.000

QOL, quality of life; mRS, modified Rankin Scale; ICU, intensive care unit.

† Statistically significant differences in clinical features and long-term outcomes between patients with better and worse QOL were tested using Fisher’s exact test for categorical data and the Mann–Whitney U test for numerical data, ^#^two patients were excluded because they did not know whether they had stayed in ICU or not, *p < 0.05.

**Figure 2:**
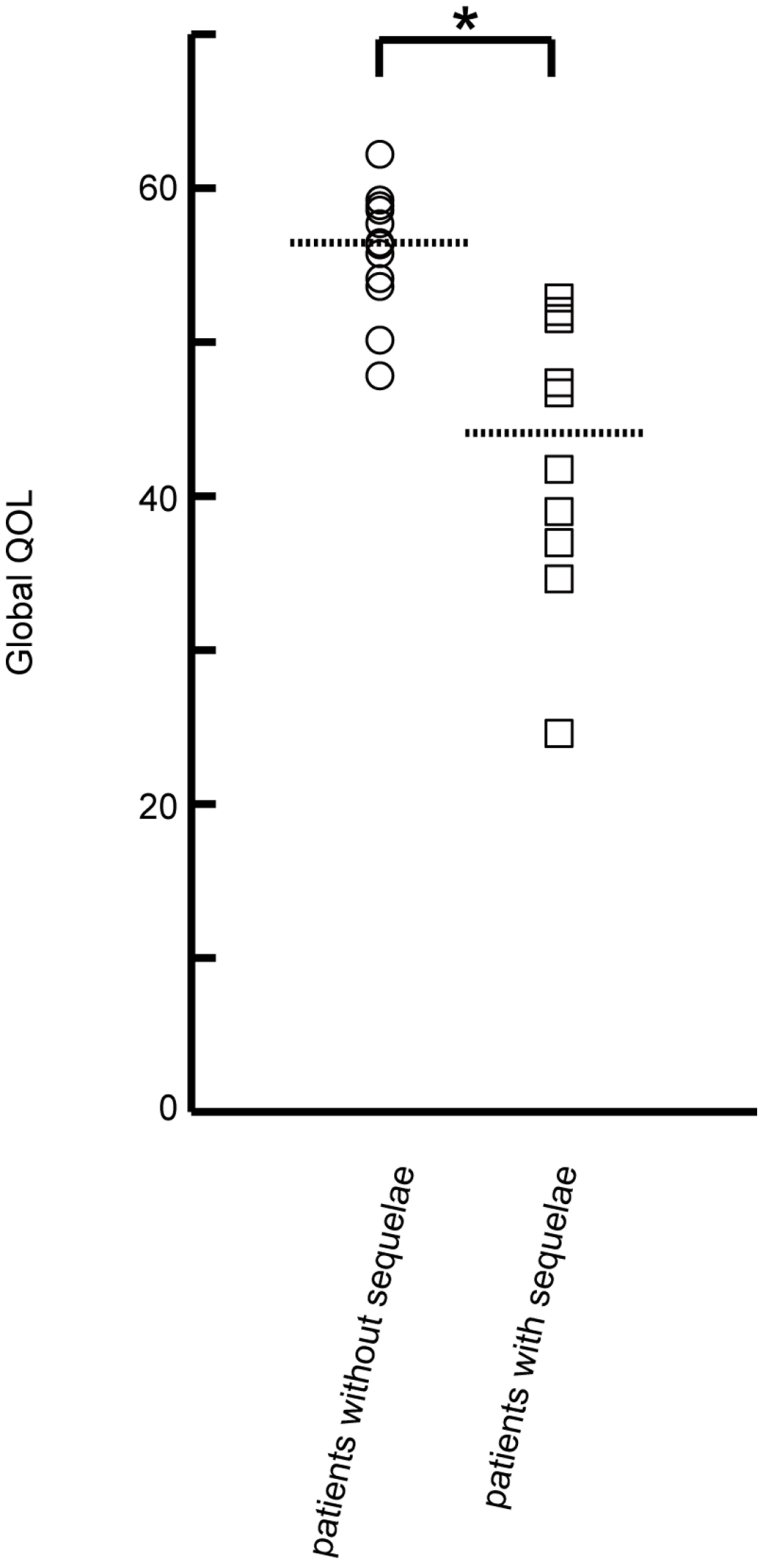
Comparison of Global QOL between patients with and without sequelae. Global QOL was calculated for each patient based on the T-scores of 12 NeuroQOL battery domains. Patients with sequelae (*n* = 10) had significantly worse Global QOL than those without sequelae (*n* = 12; 44.2 vs. 56.4, *p* < 0.001). Circles indicate the Global QOL of patients without sequelae; squares indicate the Global QOL of patients with sequelae. The dotted lines indicate the median Global QOL for patients with and without sequelae. Statistically significant differences in Global QOL were detected between patients without and with sequelae using the Mann–Whitney *U*-test: *p < 0.001.

We then compared NeuroQOL T-scores between patients with and without sequelae to confirm how these sequelae affect QOL. Patients with sequelae reported significantly worse Global QOL than those without (*p* < 0.001; Figure 2) and had significantly worse QOL for 11/12 NeuroQOL domains (Supplementary Table 3). Moreover, patients with sequelae had significantly worse Global QOL than controls (*p* = 0.037). Additional analyses revealed that patients without sequelae were more likely to return to their pre-disease state (100% vs. 40%, *p* = 0.003) and more independent at home (100% vs. 50%, *p* = 0.010; Supplementary Table 4).

## 4. Discussion

Among patients with NMDARE 6 years after onset, 46% experienced sequelae, and only 73% returned to their previous lives. Patients’ social QOL was worse than controls, especially in patients with sequelae. Furthermore, patients with sequelae demonstrated worse Global QOL than controls.

Most patients with NMDARE achieve neurologically favorable long-term outcomes, as revealed by a previous large cohort study using mRS (3), which was confirmed in our findings (Supplementary Table 1). Despite the favorable long-term mRS outcomes, early studies reported neuropsychological impairments lasting for years (17, 18), and more recent studies found that ≥60% of patients with NMDARE demonstrated sequelae 4–5 years post-disease onset (4, 6–8, 10), consistently with our findings (Supplementary Table 1). Memory disorders are reportedly more common than other sequelae (4, 6–8), particularly working memory impairments (4, 19). Similarly, memory disorders were most frequent in our study (Supplementary Table 1), although our patients were not formally diagnosed. Of the eight patients with persistent memory sequelae, only three (38%) felt that their memory skills necessary for learning new tasks or instructions were severely impacted. Regarding association of relapses with long-term outcome, a study of pediatric onset NMDARE revealed no difference in neurological long-term outcomes between patients with and without relapses (20), consistently with our findings (Table 2; Supplementary Table 4). The pathophysiological mechanisms underlying persistent sequelae in patients with NMDARE include reduced functional connectivity (21), hippocampal or cerebellar atrophy (22, 23), and white matter damage (24). Although we observed sequelae 6 years after onset, the underlying mechanisms could not be determined for lack of laboratory, imaging, or physiological data.

A prospective longitudinal study revealed improved cognition 2–5 years post-disease onset (4). Our previous study (data collection at 4 years before the current study) that examined the long-term outcomes of patient members of the Japanese Anti-NMDARE Patients’ Association found that, by 59 months, 62% demonstrated persistent sequalae (8). Comparison of our previous study (8) and our present study yields a 16% reduction in the number of patients with sequelae, possibly reflecting the natural post-NMDARE course in 60–80 months after onset, though the patient cohorts differed.

Patient-oriented outcomes are increasingly used in clinical neurology because of their direct relevance to patients (14). Narayanaswami introduced the NeuroQOL to measure patient-reported outcomes in pediatric and adult patients with neurological conditions (13, 14). The consistency and reproducibility of NeuroQOL were previously validated in various patient disease cohorts (25–27), and it has been utilized in several diseases, including multiple sclerosis (28), myasthenia gravis (29), and Parkinson’s disease (30). Importantly, this was the first study to use the NeuroQOL battery to examine patients with NMDARE. A recent report of QOL in pediatric NMDARE patients used the PedsQL inventory and found that patients 31 months post-disease onset had worse QOL than controls (9); this result was strongly correlated with fatigue. The present study is the first report to demonstrate worse QOL—especially for social domains—of adult patients with NMDARE.

McKeon’s study first demonstrated deficits in social functioning during the post-acute-phase in patients with NMDARE, evidence of impaired performance on social cognition tasks (31). Subsequent studies revealed deficits in social functioning (5–7, 32); for example, younger age at onset was associated with worse long-term adaptive behaviors, independent of neurologic disability, as evaluated by the Adaptive Behavior Assessment System (7). Blum et al. administered the Patient-Reported Outcomes Measurement Information System Psychosocial Impact Illness questionnaire to patients with NMDARE (6). Those with the worst psychosocial outcomes tended to demonstrate psychiatric comorbidities or persistent sequelae (6), to which we observed similar results (Table 2). Additionally, social domain deficits in patients with NMDARE could also be explained by their inability to resume their pre-disease activities like work and school (60%–75%) 4 or more years post-disease onset, according to this and prior studies (6, 8, 10). Importantly, initial misdiagnosis of NMDARE was associated with a decreased likelihood of returning to work/school after the acute illness phase (6). Conversely, following up with a psychiatrist after hospitalization increased patients’ odds of returning to work/school (6). Rehabilitation and social care in the post-acute-phase might positively influence long-term outcomes and increase patients’ QOL.

Patients with any sequelae experienced worse QOL than controls, particularly in social domains (Tables 1, 2). Factors that can lead to cognitive impairment, worse neurological outcomes, and reduced likelihood of returning to work/school include initial misdiagnosis, delayed treatment, and lack of second-line immunotherapy in patients refractory to first-line immunotherapy during the acute disease phase (3, 4, 6, 33). All collected data in our study were anonymized, and we could not access the details of treatments during the acute phase. However, considered alongside existing evidence, our study’s results support the use of intense interventions in the acute-phase to prevent sequelae and maximize QOL in patients with NMDARE. In other words, rapid diagnosis and appropriate treatment can ameliorate neurological outcomes, decrease sequelae, increase the likelihood of resuming prior activities, and improve long-term QOL. Therefore, adult NMDARE treatment guidelines, as that of pediatric patients (34), are urgently needed to improve these patients’ QOL.

The present study had two main limitations that should be considered. The questionnaire response rate was relatively low (39%), resulting in a relatively small patient cohort (*n* = 22), potentially leading to sampling bias and hindering multivariable analysis. However, most clinical characteristics and long-term outcomes—including neurological outcomes, sequelae, and return to daily life activities—were consistent across studies. Therefore, the effect of sampling bias appears minimal at best. Thus, we feel comfortable discussing the significance of our results, including findings regarding patients’ social domain QOL. We also lacked length of acute phase, treatments, laboratory, imaging, and physiological data. Consequently, we cannot determine factors contributing to these patients’ long-term sequelae and worse QOL.

## 5. Conclusion

Although most patients with NMDARE achieved neurologically favorable outcomes, around half experienced persistent sequelae, and fewer than three-quarters returned to their prior work/school activities. Overall, patients with NMDARE appeared similar to controls, except those who demonstrated persistent sequelae; however, social domain QOL was markedly worse. Therefore, intensive interventions to prevent sequelae in the acute-phase may improve patients’ long-term QOL for years post-disease onset.

## Data availability statement

The original contributions presented in the study are included in the article/Supplementary material, further inquiries can be directed to the corresponding author.

## Ethics statement

The studies involving human participants were reviewed and approved by Nihon University School of Medicine’s Ethics Committee. The patients/participants provided their written informed consent to participate in this study.

## Author contributions

SH and MH: study design. SH, MH, YY, and HN: data collection and writing-review and editing. SH: data analysis and writing original draft. All authors contributed to the article and approved the submitted version.

## Funding

This work was supported in part by MHLW grant number 22HA1003 and JSPS KAKENHI grant number JP20K07875 (MH).

## Conflict of interest

The authors declare that the research was conducted in the absence of any commercial or financial relationships that could be construed as a potential conflict of interest.

## Publisher’s note

All claims expressed in this article are solely those of the authors and do not necessarily represent those of their affiliated organizations, or those of the publisher, the editors and the reviewers. Any product that may be evaluated in this article, or claim that may be made by its manufacturer, is not guaranteed or endorsed by the publisher.

## Abbreviations

ICU: Intensive care unit
mRS: Modified Rankin Scale
NMDARE: Anti-N-methyl-D-aspartate receptor encephalitis
QOL: Quality of life
