# Supplementary material for: Long-term effects of anti-N-methyl-d-aspartate receptor encephalitis on quality of life
Source: Front Neurol. 2023 May 18;14:1170961. doi: 10.3389/fneur.2023.1170961 (PMC10232987; doi:10.3389/fneur.2023.1170961)
Supplement: Supplementary file 1 [file Data_Sheet_1.pdf]

Supplementary Material

Long-term effects of anti-N-methyl-D-aspartate receptor encephalitis on quality of life

Satoshi Hirose, Makoto Hara*, Yuki Yokota, Hideto Nakajima

*** Correspondence:** Makoto Hara: hara.makoto@nihon-u.ac.jp

1 Supplementary Methods

1.1 The Japanese Anti-*N*-methyl-D-aspartate Encephalitis (anti-NMDARE) Patients' Association

Japanese Anti-NMDARE Patients' Association members are patients diagnosed with definite NMDARE based on clinical symptoms and anti-NMDAR IgG in CSF, serum, or both. Family members and medical personnel are also members of the association which seeks 1) to disseminate disease-related information, 2) to promote communication between patients and family members, 3) to share study results, and 4) to increase public support for patients with NMDARE. More information can be found at https://kounmdajnouen.cloud-line.com.

1.2 Details of Questionnaire Part 1: clinical features and long-term outcomes

Structured questionnaires were distributed to patients and their families through the Japanese Anti-NMDARE Patients' Association. The questionnaires consisted of two parts: Part 1 assessed clinical features and long-term outcomes, asking the following queries, and Part 2 assessed QOL.

Query 1. What is your age at present?

Query 2. What is your sex?

1) male, 2) female.

Query 3. When did your symptoms of anti-NMDARE start?

Query 4. Were any tumors found?

1) Yes, and it was an ovarian teratoma. 2) Yes, and it was a tumor other than ovarian teratoma. 3) No, not found.

Query 5-1. Which was your worst status during the clinical course?

1) no symptoms at all, 2) no significant disability despite symptoms, able to carry out all usual duties and activities, 3) unable to carry out all previous activities, but able to look after own affairs without assistance, 4) requiring some help, but able to walk without assistance, 5)

unable to walk without assistance, and unable to attend to own bodily needs without assistance, 6) bedridden, incontinent, and requiring constant nursing care and attention, 7) dead.

Query 5-2. Which is your present status?

(the same alternatives as Query 5-1.)

Query 6. Were you admitted to an ICU?

1) yes, 2) no.

Query 7. Did you require mechanical ventilation?

1) yes, 2) no.

Query 8-1. Do you presently experience symptoms?

Query 8-1-1. If yes, for Query 8-1, which symptoms do you have?

1) psychiatric symptoms, 2) memory, 3) speech, 4) seizures, 5) movement disorders, 6) paralysis, 7) dysgeusia, 8) olfactory disorder, and 9) urinary disorders.

Query 8-2. Has your personality changed over the disease course?

Query 9. Have you returned to your previous work or school life?

Query 10. Do you have any disability or handicap in your home life at present?

Query 11. Did you have clinical relapses?

Query 11-1. If yes for Query 11, how many relapses did you have?

1.3 Definition of Global QOL

To assess the overall QOL of patients based on NeuroQOL, the 12 domain T-scores were merged into a single “Global QOL” score for each patient according to the following procedures. First, the domains were classified into two categories; one was the “positive category,” where higher T-scores indicate better QOL, and the other was the “negative category,” where higher T-scores indicate worse QOL. The positive categories included upper extremity function, lower extremity function, positive affect & well-being, cognitive function, satisfaction with social roles & activities, and ability to participate in social roles & activities; higher T-scores of these domains indicate better QOL (1). The negative category included fatigue, sleep disturbance, depression, anxiety, stigma, and emotional & behavioral dyscontrol; higher T-scores indicated worse QOL (1).

Next, we transformed T-scores in the negative category into "inverted T-scores" so that higher scores indicated better QOL. Then, positive category and negative category inverted T-scores were averaged across physical, mental, and social domains to create a "Global QOL.” For the six "negative" domains, we defined "inverted T-scores" as 100 minus the original T-score. Higher scores in inverted T-scores indicate better QOL (similar to the original T-scores of positive domain categories).

Controls' average (i.e., 50) and variance (i.e., 10) were preserved before and after the T-score transformations. Then, the 12 domains were sub-divided into physical, mental, and social domains according to the “Neuro-QOL Adult Domain Framework” (National Institute of Neurological Disorders and Stroke User Manual for the Quality of Life in Neurological Disorders (Neuro-QoL) Measures, Version 2.0, March 2015). Positive domain T-scores and inverted negative domain T-scores were averaged across the various physical domains (i.e., upper extremity function, lower extremity function, fatigue, and sleep disturbances; Supplementary Figure 1). Similarly, positive and negative inverted T-scores were averaged for mental and social domains (Supplementary Figure 1). “Global QOL” was the average of the physical, mental, and social domain scores (Supplementary Figure 1). This transformation allowed direct comparisons of each patient's overall QOL.

2 Supplementary Figures

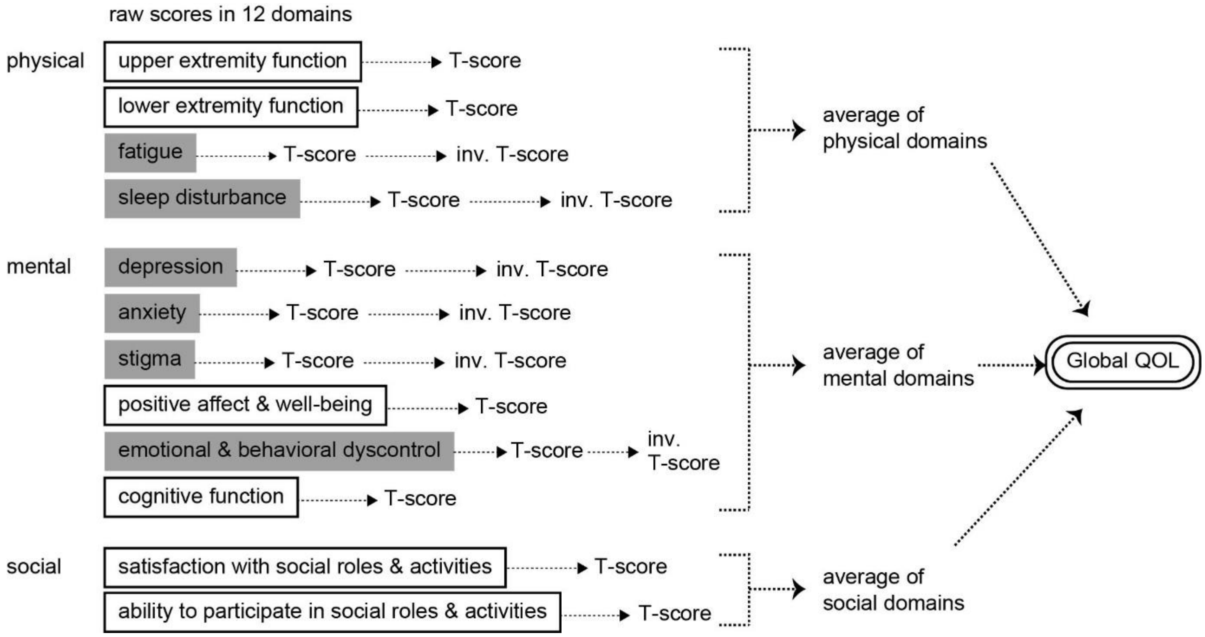

Supplementary Figure 1. Definition of Global QOL. To assess patients' overall QOL, the 12 NeuroQOL domain T-scores were merged into a single "Global QOL" score. First, the domains were classified into two categories; one was the “positive category,” where higher T-scores indicate better QOL (open rectangles), and the other was the "negative category," where higher T-scores indicate worse QOL (filled rectangles). Next, we transformed T-scores in the negative category into "inverted T-scores" so that higher scores indicated better QOL. Then, positive category and negative category inverted T-scores were averaged across physical, mental, and social domains. "Global QOL" is the average of the physical, mental, and social domain averages (depicted by a double-rounded rectangle).

3 Supplementary Tables

3.1 Supplementary Table 1. Demographics, clinical features, and long-term outcomes of patients with NMDARE

Age, y, median (range)	28 (19 - 57)
Female, n (%)	19 (86.4)
Duration since disease onset, months, median (range)	77.5 (26 - 162)
Tumor, n (%)	10 (45.5)
mRS at worst, median (range)	5 (2-5)
Stay in ICU[#], n (%)	14 (70.0)
Use of ventilator, n (%)	11 (50.0)
mRS at present, median (range)	0 (0-5)
Sequelae, n (%)	10 (45.5)
Personality change, n (%)	9 (40.9)
Return to previous work/school life, n (%)	16 (72.7)
Self-reliance at home life, n (%)	17 (77.3)
Relapse, n (%)	5 (22.7)

NMDARE: anti-*N*-methyl-D-aspartate receptor encephalitis, mRS modified Rankin Scale, ICU: intensive care unit

[#]two patients were excluded because they did not know whether they had stayed in ICU or not

3.2 Supplementary Table 2. NeuroQOL T-scores in each 12 domains of NMDARE patients

NeuroQOL domain	median (range)
Physical	
Upper extremity function#	53.8 (12.8 - 53.8)
Lower extremity function#	58.6 (16.5 - 58.6)
Fatigue†	41.8 (29.5 - 74.1)
Sleep disturbance†	40.4 (32.0 - 80.2)
Mental	
Depression†	40.0 (36.9 - 75.0)
Anxiety†	47.7 (36.4 - 76.8)
Stigma†	45.7 (39.2 - 73.7)
Positive affect & well-being#	49.0 (26.3 - 68.0)
Emotional & behavioral dyscontrol†	46.7 (32.2 - 68.1)
Cognitive function#	44.9 (17.3 - 64.2)
Social	
Satisfaction with social roles & activities#	47.9 (32.6 - 60.5)
Ability to participate in social roles & activities#	45.8 (24.1 - 60.2)

#higher scores indicate better, †higher scores indicate poorer

3.3 Supplementary Table 3. Comparison of NeuroQOL T-scores between patients with/without any sequelae

NeuroQOL domain	pts without sequelae (n = 12)	pts with sequelae (n = 10)	*p-value*[§] (comparison)
Global QOL#	56.4 (47.8 - 62.2)	44.2 (24.6 - 52.8)	<0.001**
Physical			
Upper extremity function#	53.8 (53.8 - 53.8)	37.5 (12.8 - 53.8)	0.017*
Lower extremity function#	58.6 (48.6 - 58.6)	40.3 (16.5 - 58.6)	0.002*
Fatigue†	35.3 (29.5 - 47.4)	47.4 (29.5 - 74.1)	0.021*
Sleep disturbance†	36.3 (32.0 - 48.9)	49.6 (32.0 - 80.2)	0.006*
Mental			
Depression†	36.9 (36.9 - 54.3)	53.6 (36.9 - 75.0)	0.025*
Anxiety†	42.1 (36.4 - 50.5)	55.5 (36.4 - 76.8)	0.001*
Stigma†	39.2 (39.2 - 50.6)	51.7 (39.2 - 73.7)	0.017*
Positive affect & well-being#	52.4 (40.2 - 68.0)	43.1 (26.3 - 56.8)	0.050
Emotional & behavioral dyscontrol†	42.9 (32.2 - 65.8)	53.9 (43.7 - 68.1)	0.014*
Cognitive function#	55.3 (39.9 - 64.2)	38.0 (17.3 - 64.2)	0.001*
Social			
Satisfaction with social roles & activities#	49.8 (46.3 - 60.5)	44.2 (32.6 - 48.2)	<0.001**
Ability to participate in social roles & activities#	50.9 (44.0 - 60.2)	37.8 (24.1 - 46.1)	<0.001**

pts: patients

[§]Statistically significant differences in NeuroQOL T-scores between patients with and without sequelae were detected using the Mann–Whitney U test, #higher scores indicate better, †higher scores indicate poorer, *$p < 0.05$, **$p < 0.001$

3.4 Supplementary Table 4. Comparison of clinical features and long-term outcomes between patients with/without any sequelae

	pts without sequelae (n = 12)	pts with sequelae (n = 10)	*p-value*† (comparison)
Age, median (range)	28 (20 - 40)	28.5 (19 - 57)	0.821
Female (%)	83.3	90.0	1.000
Tumor (%)	41.7	50.0	1.000
Duration since onset, months, median (range)	89.5 (26 - 162)	76 (28 - 136)	0.539
Stay in ICU# (%)	63.6	77.8	0.642
Use of ventilator (%)	33.3	70.0	0.198
Favorable mRS (≤2) at present (%)	100.0	60.0	0.029*
Return to previous work/school life (%)	100.0	40.0	0.003*
Self-reliance at home life (%)	100.0	50.0	0.010*
Relapse (%)	25.0 (%)	20.0 (%)	1.000

pts: patients, mRS modified Rankin Scale, ICU: intensive care unit

†Statistically significant differences in clinical features and long-term outcomes between patients with better and worse QOL were tested using Fisher's exact test for categorical data and the Mann–Whitney U test for numerical data, #two patients gave no answer for this item, *$p < 0.05$

4 Supplementary References

1. Cella D, Lai JS, Nowinski CJ, Victorson D, Peterman A, Miller D et al. Neuro-QOL: brief measures of health-related quality of life for clinical research in neurology. Neurology (2012) 78:1860-1867. https://doi.org/10.1212/WNL.0b013e318258f744
